# Effects of the association between *APOE* rs405509 polymorphisms and gene-environment interactions on hand grip strength among middle-aged and elderly people in a rural population in southern China

**DOI:** 10.1186/s13018-021-02522-2

**Published:** 2021-06-11

**Authors:** Haoyu He, Huaxiang Lu, Shuzhen Liu, Jiansheng Cai, Xu Tang, Chunbao Mo, Xia Xu, Quanhui Chen, Min Xu, Chuntao Nong, Qiumei Liu, Junling Zhang, Jian Qin, Zhiyong Zhang

**Affiliations:** 1 School of Public Health, Guangxi Medical University, 22 Shuangyong Road, Nanning, Guangxi Zhuang Autonomous Region China; 2 Department of Quality Management, The Affiliated Hospital of Stomatology, Guangxi Medical University, 10 Shuangyong Road, Nanning, Guangxi Zhuang Autonomous Region China; 3 Department of Guangxi Science and Technology Major Project, Guangxi Zhuang Autonomous Region Center for Diseases Control and Prevention, 18 Jinzhou Road, Nanning, Guangxi Zhuang Autonomous Region China; 4 School of Public Health, Guilin Medical University, 20 Lequn Road, Guilin, Guangxi Zhuang Autonomous Region China

**Keywords:** Hand grip strength, Apolipoprotein E, Single nucleotide polymorphism, Gene-environment interaction

## Abstract

**Background:**

Hand grip strength is a complex phenotype. The current study aimed to identify the effects of the association between *APOE* rs405509 polymorphisms and gene-environment interactions on hand grip strength among middle-aged and elderly people in a rural population in Gongcheng, southern China.

**Methods:**

*APOE* rs405509 polymorphisms in 1724 participants (695 men and 1029 women, aged 45–97 years old) were genotyped using the Sequenom MassARRAY platform. Statistical analysis was conducted using SPSS 21.0 and Plink 1.90.

**Results:**

The *APOE* rs405509 G allele was associated with lower hand grip strength in all participants (β = −1.04, *P* value <0.001), and the correlation seemed to be even stronger among women. A significant gene-environment interaction was observed between *APOE* rs405509 and smoking, especially in men. The hand grip strength of male smokers carrying the GG genotype was significantly higher than that of nonsmokers (*P* value = 0.004).

**Conclusions:**

*APOE* rs405509 polymorphisms might be genetic factors that affect hand grip strength in a rural population in Gongcheng, southern China. The *APOE* rs405509-smoking interaction has an impact on hand grip strength.

## Introduction

Hand grip strength is an indicator that can be used to characterize overall muscle strength to some extent [1]. Given that hand grip strength can be conveniently and accurately measured, it is a common component of physical examinations in clinical practice. Hand grip strength is associated with ageing, cognitive level, mental health, disease, death, and rehabilitation [2–6]. In the field of surgery, a decline in hand grip strength is associated with an increased risk of fracture at the distal forearm, vertebrae, hip, and so on [7, 8]. Hand grip strength may also be used as a predictor to evaluate the outcomes of orthopaedic surgery and fracture surgery [9, 10]. Therefore, hand grip strength is an effective indicator of the general health condition and has clinical significance.

As a complex phenotype, hand grip strength is likely influenced by multiple genetic and environmental factors [11]. The heritability of hand grip strength is estimated to be 56% [12]. Genes related to muscle function, such as vitamin D receptor, ACTN3, and UCP3 [13–15], are reportedly associated with hand grip strength.

Apolipoprotein E (*APOE*) is well known for its role in lipid metabolism. It is also essential for nervous system development, neuroprotection, synaptic plasticity regulation, and muscle innervation [16]. A study with a large sample size of 379,000 participants [17] and a systematic review [18] suggested that *APOE* is associated with muscle strength. Batterham et al. [19] provided data on the relationship between *APOE* and hand grip strength in Australians aged ≥70 years. However, insufficient evidence supports the supposition that *APOE* is associated with hand grip strength in healthy middle-aged and elderly Chinese individuals.

rs405509, an *APOE* promoter single nucleotide polymorphism (SNP), influences the expression of APOE [20]. The G allele is reportedly associated with modifying plasma lipid levels [21], causing probable dementia [22], and increasing the risk of Alzheimer’s disease [23]. A previous study showed that hand grip strength is negatively correlated with cognitive function among middle-aged and elderly Chinese individuals [24]. Nevertheless, few studies have examined the relationship between rs405509 and hand grip strength.

The study of gene polymorphisms can be classified as one kind of research in the laboratory. According to Translational Medicine, laboratory research has the potential to help clinicians and patients better cope with disease, by preventing disease, improving treatment and promoting rehabilitation [25]. Therefore, this study may have some potential significance for clinical practice.

Few studies have investigated the genetic factors that affect grip strength in rural populations in southern China. This study chose the middle-aged and elderly rural population in Guangxi Zhuang Autonomous Region (an area where ethnic minorities are concentrated in southern China) as the study population.

## Materials and methods

### Sample

A total of 1724 participants were recruited from Limu and Lianhua, Gongcheng, a county in Guilin, Guangxi Zhuang Autonomous Region, People’s Republic of China. The age of the participants ranged from 45 to 97 years, with a mean ± standard deviation (SD) age of 63.54 ± 9.81 years. All participants gave written informed consent. The participants were interviewed using a standard questionnaire to obtain demographic characteristics and lifestyle information such as a history of cigarette smoking and alcohol consumption, living habits, and level of physical activity.

All measurements were collected by trained research staff from Guangxi Medical University in accordance with a standardized protocol.

The study was approved by the institutional research ethics committee of Guangxi Medical University in 2018.

### Anthropometric measurements

A handheld dynamometer was used to measure hand grip strength. The subject was required to stand with the arm naturally straight. The dominant hand was tested three times, and the maximum value was used in the subsequent analyses. A multifunctional body weight scale was used to measure height and weight. Body mass index (BMI) was calculated using weight and height.

### DNA extraction and genotyping

Blood samples were collected from the participants by professional nurses. Genomic DNA was prepared from 1 mL of a blood sample by using a TIANamp Blood DNA kit (Tiangen, Beijing, China). SNP genotyping was conducted by Bio Miao Biological Technology Co., Ltd. (Beijing, China) by using the Sequenom MassARRAY matrix-assisted laser desorption ionization time-of-flight mass spectrometry platform (Sequenom Inc., San Diego, CA, USA).

### Statistical analysis

Age, hand grip strength, height, weight and BMI are continuous variables, presented as the mean ± SD. Sex, ethnicity, smoking, drinking, housework, farm work and SNP genotypes are categorical variables, presented as percentages. Continuous variables were compared by t-tests or variance analysis, whereas categorical variables were compared by chi-square tests. Linear regression was used to explore the association between *APOE* rs405509 genotypes and hand grip strength, adjusting for sex, age, ethnicity and height. A general linear model was used to evaluate gene-environment interactions. Once a significant interaction was observed, a simple main effect analysis was applied to estimate and compare the marginal means of hand grip strength of the interaction. T-tests, variance analyses, chi-square tests, general linear models and simple main effect analyses were conducted using the statistical software package SPSS 21.0 (SPSS Inc., Chicago, IL). Hardy–Weinberg equilibrium (HWE), minimal allele frequency (MAF) and linear regression were performed using PLINK 1.90. *P* < 0.05 was considered statistically significant.

## Results

The demographic characteristics of the study participants are summarized in Table 1. The men (*n* = 695, 40.3%) and women (*n* = 1029, 59.7%) had an average age of 64.78 ± 9.41 and 62.71 ± 9.98 years, respectively. The hand grip strength of the men was 27.01 ± 8.48 kg, which was higher than that of the women. The men were taller and heavier and have higher rates of smoking and drinking than the women. The women performed more housework than the men (Table 1).

**Table 1:** Demographic characteristics of the study participants

Variables	Mean ± SD/n (%)			*P* value
	All (*n*=1724)	Men (*n*=695)	Women (*n*=1029)	
Age (years)	63.54 ± 9.81	64.78 ± 9.41	62.71 ± 9.98	<0.001
Sex (male/female %)	40.3/59.7			
Ethnicity (Yao/Han/others %)	69.1/24.3/6.6	71.2/23.0/5.8	67.6/25.2/7.2	0.062
Hand grip strength (kg)	22.29 ± 8.07	27.01 ± 8.48	19.10 ± 5.95	<0.001
Height (cm)	154.20 ± 8.29	160.48 ± 6.40	149.96 ± 6.56	<0.001
Weight (kg)	53.72 ± 9.82	58.47 ± 9.31	50.51 ± 8.81	<0.001
BMI (kg·m^−2^)	22.53 ± 3.40	22.68 ± 3.16	22.43 ± 3.55	0.148
Smoking	323 (17.7%)	317 (45.6%)	6 (0.6%)	<0.001
Drinking	578 (33.5%)	376 (54.1%)	202 (19.6%)	<0.001
Housework	1440 (57.9%)	469 (67.5%)	971 (94.4%)	<0.001
Farm work	999 (33.5%)	412 (59.3%)	587 (57.0%)	0.356

All DNA samples were successfully genotyped, and the genotyped polymorphisms were consistent with HWE. The MAF of rs405509 was 0.347, greater than 0.05 (Table 2).

**Table 2:** Descriptive statistics of the rs405509 genotype

Gene	SNP	Alleles	Region	MAF	*P*_HWE_
*APOE*	rs405509	T/G	Promoter	0.347	0.222

The difference in the frequency of *APOE* rs405509 genotypes between the sexes was not statistically significant (*x*^*2*^ = 4.748, *P* value = 0.093). ANOVA revealed that hand grip strength was significantly different among *APOE* rs405509 genotypes in all participants and was higher in men than in women. Linear regression showed that *APOE* rs405509 was associated with hand grip strength in all participants (β = −1.04, *P* value <0.001). After adjusting for sex, age, ethnicity and height, the results showed that this genotype was associated with hand grip strength in both sexes: men, β = −0.86, *P* value = 0.038; women, β = −1.12, *P* value <0.001. The participants with the GG genotype had the lowest hand grip strength (Table 3).

**Table 3:** Association between *APOE* rs405509 genotypes and hand grip strength

Group	SNP genotypes	Frequency n (%)	Hand grip strength		β±SE	*P* value
			Mean ± SD (kg)	*P* value		
All	TT	745 (43.2)	23.11	<0.001	− 1.04 ± 0.22	<0.001
	GT	759 (44.0)	22.02			
	GG	220 (12.8)	20.43			
Males	TT	305 (43.9)	27.50	0.070	− 0.86 ± 0.41	0.038
	GT	316 (45.5)	27.03			
	GG	74 (10.6)	24.97			
Females	TT	440 (42.8)	20.07	<0.001	− 1.12 ± 0.24	<0.001
	GT	443 (43.1)	18.45			
	GG	146 (14.2)	18.13			

**Fig. 1:**
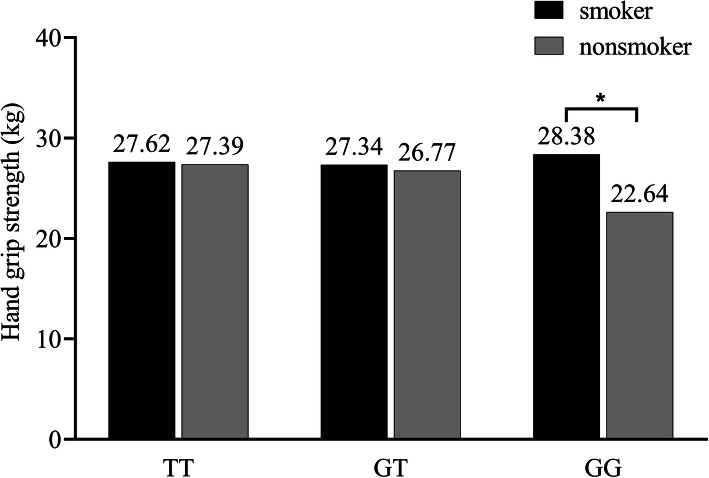
Estimated marginal means of hand grip strength of the interaction between *APOE* rs405509 and smoking in men. *P value = 0.004

Generalized linear models were used to explore the effects of the interaction between *APOE* rs405509 and environmental factors (i.e. age, sex, ethnicity, weight, history of smoking and drinking and contribution to housework and farm work) on hand grip strength. After adjusting for sex, age, ethnicity, height, presence of rs405509 and history of smoking, the results showed that the interaction between *APOE* rs405509 and smoking history was significant (*P* value = 0.021), especially among men (*P* value = 0.007) (Table 4). To further understand the effects of the interaction, we used a simple main effect analysis to explore the estimated marginal means of hand grip strength. Given that only six women among all of the participants had a history of smoking, only the men who smoked were analysed. The hand grip strength of smokers with the GG genotype was significantly higher than that of nonsmokers (*P* value = 0.004). Among those with the TT and GT genotypes, the difference in hand grip strength between smokers and nonsmokers was not statistically significant (Fig. 1).

**Table 4:** Analysis of the interaction between *APOE* rs405509 and smoking

Group	Sum of squares	df	Mean square	F	*P* value
All	299.259	2	149.630	3.853	0.021
Males	509.224	2	254.612	4.990	0.007
Females	35.687	1	35.687	1.250	0.264

## Discussion

To determine the relationship between the *APOE* rs405509 polymorphism and hand grip strength, we recruited middle-aged and elderly participants from two towns in Guilin, a world-famous tourist city. Given that the traffic situation in the area is inconvenient, the participants live in a relatively isolated environment and all follow similar customs.

The results showed that the G allele of *APOE* rs405509 was significantly correlated with a lower hand grip strength. APOE is well known for its role in the production, conversion, and clearance of lipids [26, 27]. APOE is also involved in the regulation of the nervous system, such as affecting synaptic plasticity, maintaining neuronal membranes, supporting physiological neurotransmission, and influencing the efficiency of multiple homeostatic pathways in the brain [28, 29]. Moreover, APOE is associated with peripheral nerve regeneration and subsequent neuromuscular junction reinnervation [30]. We hypothesized that the effects of the *APOE* rs405509 G allele on neurological function are ultimately related to the muscle strength of grip. Furthermore, *APOE* is closely related to ageing-related phenotypes, including physical decline, diseases, and longevity. *APOE* may play an important role in an ageing-related pathway [17]. Lu et al. [31] found that the T allele of *APOE* rs405509 is associated with longevity in a population of Han Chinese individuals. Skoog et al. [32] supported the results of the present study. They found that Swedes aged 79 years who are noncarriers of the *APOE* ɛ4 allele have a higher hand grip strength than those who carry this allele. A difference between this study and the present work was that our study population recruited not only elderly people but also middle-aged people. However, Alfred et al. [33] and Vasunilashorn et al. [34] did not find a relationship between *APOE* and hand grip strength among middle-aged and elderly people. These conflicting results may be caused by the heterogeneity of different study populations. The effect of *APOE* on hand grip strength may also vary due to underlying genetic heterogeneity. Thus, further research is needed to replicate the association between *APOE* and hand grip strength in sufficiently powered samples and other populations.

The association between *APOE* rs405509 polymorphisms and hand grip strength seemed to be stronger in women than in men. Hand grip strength is influenced by genetic and environmental factors. Hand grip strength can be improved by training [35, 36]. In this study, all participants came from two towns in Gongcheng, an agricultural region famous for growing persimmons. Their main source of income is the cultivation and sale of persimmons and food crops. Men are responsible for heavy physical labour, such as carrying water and tilling the soil. These activities are equivalent to long-term muscle training. Therefore, environmental factors probably had a greater influence on hand grip strength in men than in women. This possibility likely diminished the effects of genetic factors observed herein.

An interesting finding of the present study was the remarkable effects of the interaction between *APOE* rs405509 and smoking. The effects of this interaction were significant in men only, likely because the sample of female smokers in this study was too small. A simple main effect analysis of the estimated marginal means of hand grip strength revealed that smokers with the GG genotype had a higher hand grip strength than nonsmokers. Thus far, no evidence can explain this result. Nevertheless, Luo et al. [37] found that younger people with a higher smoking rate have a higher hand grip strength than older people with a lower smoking rate. Since smoking can lead to cardiovascular disease [38], lung disease [39] and other diseases [40], older people who smoke for a long time are more likely to experience disease. Older people tend to quit smoking at the time of physical and functional decline [41]. A similar point was noted by Wang et al. [42], who suggested that current smokers may be healthier and thus have a higher hand grip strength. In this study, nonsmokers with the GG genotype may have relatively poor health, which led to this result. This hypothesis must be validated in another study with a larger sample size and focus on the relationship between health and *APOE* rs405509 polymorphisms.

*APOE* is a well-known gene related to many diseases and is also being noticed in the field of surgery. Hand grip strength is a phenotype that reflects general muscle strength. It is associated with bone mineral density [37] and dynamic body balancing ability [43]; thus, a low hand grip strength is a risk factor for falls and fractures [8]. Hand grip strength can also be a predictor of surgical outcomes [9, 10]. Based on the clinical significance of grip strength and the effect of genetic variations in *APOE* on grip strength, we suspect that *APOE* rs405509 polymorphisms might serve as one of the genetic markers for predicting diseases and the risk of fracture associated with lower grip strength. Hence, in future research, we will explore the role of *APOE* variations in muscle strength and its relationship with fracture and other diseases, providing some basis for prevention. Guang-Rong Ji’s study on the vitamin D receptor gene would be a valuable reference for our future research [44].

To the best of our knowledge, the present study is one of the few to explore the association between *APOE* rs405509 polymorphisms and hand grip strength among middle-aged and elderly people in a rural population in southern China. The results showed that the interaction between *APOE* rs405509 and smoking has an influence on hand grip strength. These findings are the major contributions of this study. However, our study has several limitations. First, a cluster sampling technique was used. Hence, this study only represented the population in one area, and the findings must be validated in other populations. Second, because of the small sample size, the results must be further confirmed by subsequent studies with larger sample sizes.

## Conclusions

In summary, *APOE* rs405509 polymorphisms are associated with lower hand grip strength among middle-aged and elderly people in a rural population in Gongcheng, Guilin, China. The interaction of *APOE* and smoking has an impact on hand grip strength. Male smokers with the *APOE* rs405509 GG genotype have higher hand grip strength. *APOE* rs405509 may be a potential genetic marker of hand grip strength. These associations should be verified in other populations with larger sample sizes. The relationship between *APOE* rs405509 polymorphisms and diseases related to hand grip strength needs further exploration.

## Data Availability

Please contact the authors for reasonable requests.
